# See & Eat! Using E-books to Promote Vegetable Eating Among Preschoolers: Findings From an Italian Sample

**DOI:** 10.3389/fpsyg.2021.712416

**Published:** 2021-08-27

**Authors:** Marcella Caputi, Katrina May Dulay, Daniela Bulgarelli, Carmel Houston-Price, Giuseppina Cerrato, Mauro Fanelli, Natalie A. Masento, Paola Molina

**Affiliations:** ^1^Department of Psychology, Università degli Studi di Torino, Torino, Italy; ^2^Sigmund Freud University, Milano, Italy; ^3^School of Psychology and Clinical Language Sciences, University of Reading, Reading, United Kingdom; ^4^Department of Education, University of Oxford, Oxford, United Kingdom; ^5^Department of Chemistry, Università degli Studi di Torino, Torino, Italy; ^6^Interuniversity Department of Regional and Urban Studies and Planning, Università degli Studi di Torino, Torino, Italy

**Keywords:** vegetable intake, visual familiarity, visual exposure, healthy eating, food fussiness, mealtime goal

## Abstract

Different strategies have been developed to help parents with introducing new or disliked vegetables. Nonetheless, many parents of preschoolers struggle against children's refusal to eat vegetables. In this study, we aimed to evaluate the effectiveness of e-books in promoting positive attitudes toward vegetables through repeated visual exposures. A total of 61 families with preschoolers joined the See & Eat study and received an e-book about one of two vegetables chosen from a list of 24. Parents provided ratings of children's willingness to taste, intake, and liking of the chosen vegetables before and after reading the e-book; parents also evaluated their children's food fussiness and their agreement with respect to three mealtime goals of the family. Using a 2 (vegetable: target or non-target) × 2 (time: pre-test or post-test) within-subjects analysis, results from 53 families revealed a significant increase in children's willingness to taste, intake, and liking at post-test of both target and non-target vegetables. Following a two-week parent-child e-book reading intervention, children's food fussiness and parents' endorsement of positive mealtime goals slightly but significantly increased. Results suggest that e-books are effective in encouraging healthy eating among preschoolers and that the positive effect of e-book reading can generalize to other vegetables.

## Introduction

Research suggests that vegetable consumption has important health benefits and can protect against a number of diseases (Holley et al., 2017). Despite these benefits, vegetable intake among children across European countries, including Italy, falls below the recommended portions per day (Yngve et al., 2005; Albani et al., 2017; World Health Organization, 2019; Rosi et al., 2021). Encouraging preschool-aged children to eat more vegetables is especially important, as food preferences develop early and can predict later dietary variety and picky eating behaviors (Coulthard et al., 2010; Cardona Cano et al., 2015; Fernandez et al., 2020). A systematic review has shown that repeated exposures to food is one of the most effective strategies to improve vegetable consumption in early childhood (Holley et al., 2017). Besides measures of intake, which describes the quantity of food that children eat, willingness to taste and liking are also considered as informative variables linked to healthy eating in preschoolers. Willingness to taste indicates children's openness to introduce new food into their diet; whereas liking could be seen as a complementary aspect to intake, as liking does not automatically lead to increased intake, yet increased intake can sometimes improve liking (Houston-Price et al., 2009b; Heath et al., 2014; Mura Paroche et al., 2017; Zeinstra et al., 2018). It is worth noting that a peak in food fussiness (i.e., eating selectively, being picky, and refusing both familiar and unfamiliar foods) is typically reported between 2 and 5 years of age; this can interfere with vegetable willingness to taste, liking, and intake among children (Addessi et al., 2005; Cooke and Wardle, 2005). Food fussiness also presents challenges for family mealtimes, as children's refusal to eat some food can raise conflicts, or prevent parents from preparing specific foods for the whole family (Snuggs et al., 2019b).

Therefore, it is not surprising that many interventions have been conducted within this critical period, both at home and in school settings (Holley et al., 2017; Matwiejczyk et al., 2018; Nekitsing et al., 2018) to improve children's vegetable consumption. Overall, different methods have been used with children and/or with their parents and have been described by systematic reviews. Among the identified techniques to encourage vegetable consumption included repeated taste exposure, food adaptations, mealtime adaptations, educational interventions, pairing the vegetable with a well-known food, giving tangible rewards when the child tastes or eats vegetables, modeling, offering a choice between two different vegetables, offering a variety of vegetables, and working on the visual presentation of vegetables to enhance their appeal (Holley et al., 2017; Nekitsing et al., 2018). Repeated taste exposures proved to be one of the most effective strategies to increase 2–5-year-olds' vegetable consumption (Holley et al., 2017; Nekitsing et al., 2018). Despite the effectiveness of this strategy, it is also challenging for parents to achieve alternative strategies to create a social environment that reinforces the acceptance of new foods (Carruth and Skinner, 2000). An alternative way to familiarize children with vegetables, without necessarily tasting them, is repeated visual exposures using vegetable picture books. These books depict the “farm to fork” journey of different vegetables so that children become visually familiar with specific foods through a rather enjoyable activity, i.e., joint reading with parents. Previous studies have demonstrated the positive impact of picture books on children's willingness to taste, intake, and liking of the vegetables seen in the books (Houston-Price et al., 2009a,b; Heath et al., 2014; Barnes and Warren, 2017; Owen et al., 2018; Houston-Price et al., 2019). Our research group developed vegetable e-books to achieve similar aims. E-book reading, just like picture book reading, is a socially rewarding activity that elicits positive feelings due to the interaction between the child and the caregiver, which in turn could encourage positive attitudes toward the vegetable featured in the book (de Droog et al., 2014). A review about screen use among children presented mixed evidence for increased engagement when books are accessed through a digital device, and caution against features of e-books that may distract, rather than enhance, the reading experience (Hassinger-Das et al., 2020). Nevertheless, e-books have several potential advantages over physical picture books; for example, mass-scale distribution at little to no cost, instant access for caregiver-child pairs who want to interact by reading the e-books together, and digital features that enable interactivity and personalisation by editing the e-book with content that is personally relevant to families who are using them (Dulay et al., 2020). The See & Eat project is making vegetable e-books available in several European countries including Italy, the United Kingdom, Poland, and Finland.

We aim to evaluate whether e-books promote positive attitudes toward vegetables in children, since visual familiarity is effective in promoting vegetable consumption (Heath et al., 2011). Our main hypothesis is that repeated visual exposures to a vegetable via an e-book will result in higher levels of willingness to taste, intake, and liking of the vegetable that children have seen in an e-book compared to a control, non-target vegetable. We also aim to explore potential secondary outcomes of visual exposure; namely, a decrease in food fussiness and a change in parents' family mealtime goals. Finally, we explore whether families make use of the interactive and personalisation features of the e-books using the *Our Story 2* app. The current paper reports on the first available data, collected among an Italian sample of families of preschoolers.

## Method

### Participants

Participants were recruited using adverts placed on social media, in the local press, in kindergartens and other places frequented by families with children (libraries, swimming pools, etc.). Adverts indicated the two essential requirements for participating in the study: having a child aged between 18–48 months and having access to an iPad or tablet device. Around 120 families expressed interest in joining the study. However, about half of them did not possess an iPad/tablet or had a model that was not compatible with the app needed to read the e-book; in this case, parents were offered the option to loan a tablet from the research team. Overall, families of 61 children (*M* age = 35.51 months, *SD* = 10.62, range = 18.85–58.36, 66% male) completed the pre-test phase of the study. The survey was mostly completed by mothers (93%), with the rest of the respondents being fathers.

Three parents withdrew from the study due to personal reasons; other five discovered only after completing the pre-test that they did not own a compatible tablet device; two parents did not read the e-books. Therefore, a total of 51 participants (84% of the pre-test sample) completed the post-test phase of the study protocol.

### Measures

#### Demographic Questionnaire

The following background information was collected: child's gender, date and place of birth, daycare attendance (and the number of meals eaten there), birth order, and any diagnosed conditions (e.g., neurodevelopmental disorders, visual impairment, etc.). Children's age in months was computed by subtracting the date of the pre-test assessment from the child's date of birth. In addition, parents were asked to report their level of education, the number of children at home, and the survey respondent's relationship to the child. Parent's level of education was reported as follows: 1—No formal qualifications, 2—CSE/O-level/GCSE/School certificate or equivalent, 3—Vocational qualifications (e.g., NVQ 1 & 2), 4—A-level/ higher school certificate or equivalent (e.g., NVQ 3), 5—Bachelor's degree or equivalent (e.g., NVQ 4), or 6—Higher degree (Masters/PhD/PGCE/Postgraduate certificate).

#### Choosing Two Vegetables

Parents chose two vegetables from a list of 24 and selected one or more of four reasons for choosing them: the child refuses to eat the vegetable; the child dislikes the vegetable; the parent would like the child to eat the vegetable more often; others in the family eat the vegetable. Ratings of willingness to taste, intake (portion size), and liking were collected with reference to the two vegetables chosen.

#### Willingness to Taste

Parents reported what the child did when they were last offered a taste of each of the two vegetables. Responses were ratings on a 6-point Likert scale (0—I have not offered it yet; 1—Refused to touch food; 2—Touched food but did not put in/near mouth; 3—Put food on lips but not in mouth; 4—Put food in mouth but spat out/did not eat; or 5—ate food).

#### Intake—Portion Size

Parents reported how much of the two vegetables the child ate when they last ate it. Responses were ratings on a 5-point Likert scale: 0—My child did not eat any; 1—A tiny taste (a nibble); 2−1 teaspoon (a bite); 3−1 dessert spoon (several bites); or 4—A child-sized portion or more (lots of bites).

#### Intake—Food Frequency

Parents reported how frequently their child had consumed a portion of 24 different vegetables in the last 2 weeks. Responses ranged from 0-4 (0—Never; 1—Once; 2—A few times; 3—Many times; or 4—Everyday). The individual ratings given for the two vegetables chosen by parents were used to evaluate the main hypothesis relating to frequency of intake.

#### Liking

Parents reported their perception of how much their child liked the two chosen vegetables. Responses were ratings on a 6-point Likert scale (0—My child did not try it; 1—Disliked it a lot; 2—Disliked it a bit; 3—Neither liked nor disliked it; 4—Liked it a bit; or 5—Liked it a lot).

#### Offers to Try the Vegetables

In the post-test phase, parents were asked if they had offered the two vegetables in the last 2 weeks. Parents responded either yes or no for each food.

#### Children's Eating Behavior Questionnaire: Food Fussiness Subscale (CEBQ:FF)

Parents rated the six statements of this subscale about children's attitudes toward food on a 5-point scale (1—Never; 2—Rarely; 3—Sometimes; 4—Often; or 5—Always) (Wardle et al., 2001). Negatively worded statements were reverse coded. A total sum was computed, with higher scores indicating greater fussiness.

#### Family Mealtime Goals Questionnaire

Nine items from three subcomponents of the Family Mealtime Goals Questionnaire were used for this study (Snuggs et al., 2019a). The three sub-components represented three goals that parents can have during mealtimes: shared family food, stress/conflict avoidance, and family involvement in mealtimes. Parents rated each of the statements with 5—Strongly Agree, 4—Agree, 3—Neither agree nor disagree, 2—Disagree, or 1—Strongly Disagree. A mean score was computed from items comprising each of the three sub-components relating to mealtime goals, with higher scores reflecting priority of goal.

#### Book Engagement

Parents were asked the following questions: (1) Did you read the e-book with your child? (Yes/No); (2) Roughly how many times did you read it? (3) On average, how long did you spend reading the story (in minutes); (4) How much did you enjoy reading the e-book with your child? (5-point response scale from 1—Not at all to 5—Very much); (5) How much did your child enjoy looking at the e-book? (5-point response scale from 1—Not at all to 5—Very much); (6) Did you edit the e-book? (Yes/No); (7) Did you add or change any of the following? (photos, text, audio, video); and (8) Was your child involved in editing the e-book? (Yes/No).

### Procedure

The research design and main confirmatory hypothesis were preregistered through the Open Science Framework (Dulay et al., 2019). Power calculations for a repeated measures ANOVA were made using G^*^Power at a level of 0.80 based on estimated effect sizes from previous studies that measured changes in the primary outcomes after repeated exposures (e.g., Heath et al., 2014). Calculations for willingness to taste and liking were set at Cohen's *f* = 0.20, yielding an estimated sample size of 52; whereas intake was set at Cohen's *f* = 0.10, which resulted in an estimated sample size of 200 to detect relatively small changes in the amount of food eaten. Having collected 53 complete post-tests, the analyses can be considered sufficient for willingness to taste and liking but underpowered for the intake measure.

Data collection occurred from October 2019 to February 2020. Parents joined the study by signing an informed consent form and providing their email address. After joining the study, participants received via email a link to the pre-test questionnaire (which was completed online via Google Forms). In the pre-test phase, families provided demographic information and responses to the CEBQ:FF, food frequency, and Family Mealtime Goals questionnaires. Families then chose two vegetables (out of 24 options) that their children had tried in the past but were not keen on. One of the two vegetables chosen was randomly assigned as the target vegetable, and the other was assigned as the non-target vegetable. Families rated children's willingness to taste, intake, and liking of these two vegetables.

After completing the pre-test questionnaire, families received a link to download the *Our Story 2* app and an e-book about the target vegetable. The e-books encompass the farm to fork journey of one vegetable, with a total of 12 pages each showing images and a simple short sentence of a step in the journey; how the vegetable grows, where it can be purchased (e.g., market, shops) and how it is cleaned, prepared, cooked, and eaten. Short sentences describe the main content of the page (for an example of the e-books in English, see Figure 1 in Dulay et al., 2020). The Italian e-books followed the same structure but some were adapted to feature vegetables, images, and recipes that were more common in the country. These were accessed via an iPad or Android tablet using the *Our Story 2* app, which enabled users to open the e-books and swipe through to move through the pages. The app contained interactive features that enabled users to create their own e-books or to add their own photo, text, audio, and video content into an existing one. All families were instructed to read the e-book every day for 2 weeks. Half of the families were told explicitly about the editing capabilities of the *Our Story 2* app and were encouraged to personalize the e-books as they wished. Nevertheless, in the following analyses all participants were treated as a unique group, since only few parents actually edited the e-books. Some families who edited the e-books belonged to the group that received additional information and prompts to edit the e-book; while other families used the editing features of the e-book spontaneously, without any explicit instructions or prompting. One week after completing the pre-test, participants were reminded by email to keep using the e-book for another week.

**Figure 1:**
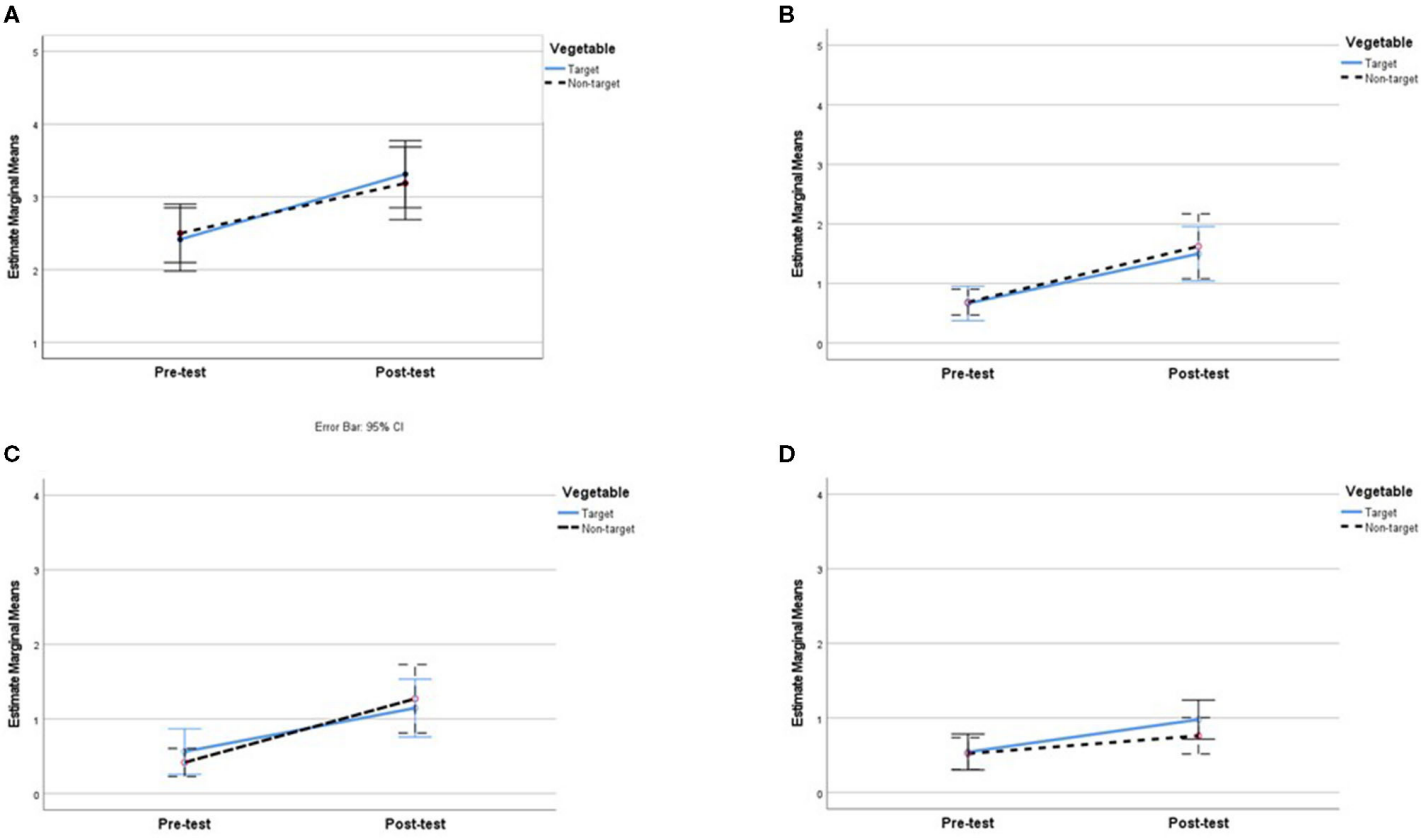
Mean reported willingness to taste, intake, and liking of the target and non-target vegetable at pre-test and post-test. **(A)** Willingness to taste (*N* = 48). **(B)** Intake (Portion size, *N* = 48). **(C)** Intake (Food frequency, *N* = 48). **(D)** Liking (*N* = 48).

After 2 weeks, participants were sent a link to a post-test survey (to be completed online via Google Forms, like the pre-test questionnaire). During the two-week reading period, parents had not been instructed to offer the target and non-target vegetable to their child. Before completing the post-test survey, they were asked if they had done so. Parents who had spontaneously offered their child both vegetables completed the post-test survey right away. Parents who had not offered their child both vegetables during the two-week reading period were encouraged to do so within the next 2 weeks. Parents who agreed to do so were sent an e-mail reminder after 1 week and a link to complete the post-test survey after 2 weeks.

In the post-test survey, parents reported theirs and their children's engagement with the e-books, and provided ratings of children's willingness to taste, intake (portion size and frequency) and liking of the vegetables offered, along with ratings of their child's food fussiness, and responses to the Family Mealtime Goals questionnaire.

## Results

Table 1 reports the participants' demographic characteristics and responses to pre-test measures. Pre-test ratings of willingness to taste, intake, and liking of the target and non-target vegetables supported parents' perceptions that children were not keen on the two vegetables, as they were neither consumed frequently nor liked by the children, on average.

**Table 1:** Demographic characteristics and scale scores at pre-test (*N* = 61).

	**Mean (SD) unless otherwise indicated**
Child age (in months)	35.51 (10.62)
Child gender (*N* male)	40
Parent 1 education	5.28 (0.94)
Parent 2 education	4.98 (1.14)
Daycare attendance (*N* yes)	54
Frequency of meals at daycare	4.94 (0.24)
*N* children at home	1.70 (0.72)
Birth order (*N* first born)	33
Target willingness to taste	2.34 (1.53)
Non-target willingness to taste	2.48 (1.48)
Target portion size	0.49 (0.96)
Non-target portion size	0.46 (0.68)
Target food frequency	0.56 (0.81)
Non-target food frequency	0.48 (0.67)
Target liking	0.64 (0.95)
Non-target liking	0.72 (0.80)
Food fussiness	17.18 (6.57)
**Family Mealtime Goals**	
Shared family food	4.2 (0.70)
Stress/conflict avoidance	4.15 (0.68)
Family involvement at mealtimes	3.71 (0.69)

Table 2 presents a summary of the vegetables chosen at pre-test, with the mean frequency of the child consuming a portion of each vegetable, and parents' reasons for choosing them. The five most frequently chosen vegetables were courgette (13 times), spinach (12), broccoli (10), fennel (9), and peas (9). The most frequently endorsed reason for choosing a vegetable was “My child refuses to eat this food” (29.27%), followed by “Others in the family eat this food regularly” (28.06%), “My child dislikes this food” (23.78%), and “I want my child to eat this food more often” (18.29%).

**Table 2:** Vegetables chosen at pre-test and average frequency of consumption (*N* = 61).

**Vegetable**	**No. of times that parents selected this vegetable**	Frequency of child consuming this vegetable (range = 0–4)^*^	**Reason for choosing the vegetable** **(no. of times that parents selected this reason)**			
		**Mean (SD)**	**Child refuses to eat it**	**Child dislikes it**	**Parents want the child to eat it more often**	**Others in the family eat it**
Artichoke	3	0.11 (0.41)	1	1	0	2
Asparagus	2	0.05 (0.22)	0	1	1	1
Aubergine	3	0.30 (0.62)	1	0	0	2
Beetroot	2	0.43 (0.74)	2	1	0	1
Broad beens	1	0.05 (0.22)	0	1	0	0
Broccoli	10	0.89 (0.86)	6	2	2	4
Brussel sprouts	4	0.05 (0.22)	2	1	1	1
Butternut squash	6	1.11 (0.99)	2	0	3	3
Cabbage	2	0.21 (0.55)	1	1	0	0
Carrots	7	1.93 (1.11)	5	0	1	1
Cauliflower	7	0.67 (0.89)	1	3	1	2
Courgette	13	1.46 (1.13)	7	4	4	7
Cucumber	1	0.18 (0.67)	0	0	0	1
Green beans	4	1.03 (0.97)	1	0	2	2
Lettuce	9	0.79 (1.10)	2	3	2	4
Leeks	1	0.28 (0.58)	0	0	0	1
Turnip	2	0.02 (0.13)	0	0	2	0
Fennel	9	0.98 (1.10)	1	4	2	4
Peas	9	1.26 (1.00)	5	1	1	2
Peppers	3	0.25 (0.54)	2	1	1	1
Spinach	12	0.95 (0.92)	5	4	5	4
Potato	4	1.84 (0.97)	1	1	3	2
Cherry tomato	5	0.89 (1.16)	1	5	0	1
Tomato	2	1.60 (1.27)	1	0	1	0

* *(0—Never; 1—Once; 2—A few times; 3—Many times; or 4—Everyday)*.

A total of 12 families modified the e-book by adding videos, photos, and/or short sentences, whether or not they received additional guidance about the editing features of the app (7 families) or did not (5 families). Guidance consisted of a small symbol that encouraged families to personalize parts of the book that depicted the shopping to food preparation steps. Families who provided a reason for not editing the e-book gave the following reasons: three parents reported that the child was not interested in editing the e-book; six parents reported time constraints; five parents reported that they felt that editing the book was not necessary; four parents did not understand that editing was required; and one parent attempted to make changes but experienced difficulties in editing the e-book. A supplemental non-parametric analysis revealed no significant differences in the primary (willingness to taste, intake, liking) or secondary (food fussiness, family mealtime goals) outcomes between families who edited the e-book and families who did not (all *p* > 0.068), suggesting that no additional benefit of editing the e-book on the study outcomes was evident from the data.

### Book Engagement and Offers to Try Vegetables

Table 3 reports responses to questions about families' engagement with the e-books engagement questions. On average, families read the e-book 8.40 times (*SD* = 5.52; range from 2 to 15; median = 9.50) for 6.88 mins (*SD* = 4.42; range from 2 to 20; median = 5.00). Parents generally rated reading the e-book as an enjoyable experience for themselves (*M* = 3.45, *SD* = 0.86) and for their children (*M* = 3.31, *SD* = 1.03).

**Table 3:** Responses to e-book engagement questions (*N* = 51).

**Question**	***M* (*SD*) unless otherwise indicated**
Did you read the e-book? (*N* yes)	51
Number of times that e-book was read	8.40 (5.52)
Average minutes spent reading the story	6.88 (4.42)
How much did you enjoy reading the e-book with your child?	3.45 (0.86)
How much did your child enjoy looking at the e-book?	3.31 (1.03)
Did you edit the e-book? (*N* yes)	12
Was your child involved in editing the e-book? (*N* yes)	10

Having access to a vegetable e-book prompted 97% of families to spontaneously offer the target vegetable to their children and 84% of families to offer the non-target vegetable. Among the 12 families who did not spontaneously offer both vegetables, 11 agreed to try offering both vegetables within the following 2 weeks. All of the 11 families who completed the second round of post-test surveys confirmed that they offered both vegetables to their child.

### Willingness to Taste, Intake, and Liking

We hypothesized that repeated exposure to a particular vegetable using an e-book would result in more positive attitudes toward that vegetable compared to a non-exposed vegetable. Changes in children's food attitudes were evaluated in a 2 (*vegetable*: target or non-target) × 2 (*time*: pre-test or post-test) repeated measures ANOVA on ratings of willingness to taste, portion size, food frequency, and liking. For families who did not spontaneously offer both vegetables during the reading period, but agreed to try offering both vegetables after this, ratings from the second post-test round were included in this analysis. The ratings given at pre-test and post-test are summarized in Table 4.

**Table 4:** Raw pre-test and post-test ratings of willingness to taste, intake, and liking (*N* = 49).

**Measure *M* (*SD*)**	**Pre-test**		**Post-test**	
	**Target**	**Non-target**	**Target**	**Non-target**
Willingness to taste	2.37 (1.52)	2.45 (1.41)	3.35 (1.59)	3.22 (1.72)
Intake (portion size)	0.55 (1.04)	0.41 (0.64)	1.20 (1.38)	1.33 (1.61)
Intake (food frequency)	0.53 (0.80)	0.53 (0.72)	0.98 (0.87)	0.79 (0.83)
Liking	0.65 (0.99)	0.67 (0.75)	1.55 (1.60)	1.67 (1.89)

#### Willingness to Taste

The results of the two-way repeated measures ANOVA (*n* = 49) revealed a significant main effect and a large effect size of time on children's willingness to taste the vegetables, *F*_(1, 48)_ = 15.06, *p* < 0.001, ${\eta_{\text{p}}}^{2}$ = 0.239. At post-test, children were more willing to put both the target vegetable (mean = 3.35) and the non-target vegetable on the lips (mean = 3.22) compared to pre-test (mean target vegetable = 2.37; mean non-target vegetable = 2.45). However, the analysis revealed neither a significant main effect of vegetable, *F*_(1, 48)_ = 0.01, *p* = 0.914, ${\eta_{\text{p}}}^{2}$ = 0.000, nor a significant interaction between vegetable and time, *F*_(1, 48)_ = 0.29, *p* = 0.595, ${\eta_{\text{p}}}^{2}$ = 0.006 (see Figure 1A).

#### Intake (Portion Size)

ANOVA results (*n* = 49) revealed a significant main effect and a large effect size of time on the portion sizes consumed by children, *F*_(1, 48)_ = 23.80, *p* < 0.001, ${\eta_{\text{p}}}^{2}$ = 0.332. At post-test, children consumed bigger portions of both the target vegetable (mean = 1.20) and the non-target vegetable (mean = 1.33) compared to pre-test (mean target vegetable = 0.55; mean non-target vegetable = 0.41). However, the analysis revealed neither a significant main effect of vegetable, *F*_(1, 48)_ = 0.00, *p* = 0.950, ${\eta_{\text{p}}}^{2}$ = 0.000, nor a significant interaction between vegetable and time, *F*_(1, 48)_ = 0.87, *p* = 0.355, ${\eta_{\text{p}}}^{2}$ = 0.018 (see Figure 1B). These results should be taken with caution as the preliminary analysis revealed that the sample size was underpowered for intake.

#### Intake (Food Frequency)

In this analysis (*n* = 47), the reported frequency of eating the target and non-target vegetable was extracted from frequency ratings of the 24 vegetables in the study. Results of the repeated measures ANOVA demonstrated a significant main effect and a large effect size of time, *F*_(1, 46)_ = 8.47, *p* = 0.006, ${\eta_{\text{p}}}^{2}$ = 0.156, with the chosen vegetables eaten more frequently at post-test (mean = 0.88) than at pre-test (mean = 0.53). However, the analysis revealed neither a significant main effect of vegetable, *F*_(1, 46)_ = 1.05, *p* = 0.310, ${\eta_{\text{p}}}^{2}$ = 0.022, nor a significant interaction between vegetable and time, *F*_(1, 46)_ = 1.25, *p* = 0.269, ${\eta_{\text{p}}}^{2}$ = 0.027 (see Figure 1C). These results should be taken with caution as the preliminary analysis revealed that the sample size was underpowered for intake.

#### Liking

At post-test, higher ratings of liking were given for both the target vegetable (mean = 1.55) and the non-target vegetable (mean = 1.67) compared to pre-test (mean target vegetable = 0.65; mean non-target vegetable = 0.67), as supported by a significant main effect and a large effect size of time on liking in the two-way ANOVA (*n* = 49), *F*_(1, 48)_ = 22.04, *p* < 0.001, ${\eta_{\text{p}}}^{2}$ = 0.315. However, the analysis revealed neither a significant main effect of vegetable, *F*_(1, 48)_ = 0.17, *p* = *0.6*83, ${\eta_{\text{p}}}^{2}$ = 0.004, nor a significant interaction between vegetable and time, *F*_(1, 48)_ = 0.09, *p* = 0.767, ${\eta_{\text{p}}}^{2}$ = 0.002 (see Figure 1D).

### Food Fussiness and Family Mealtime Goals

Paired samples *t-*tests (*n* = 47) were conducted to evaluate changes in children's food fussiness and three dimensions of family mealtime goal (shared family food, stress/conflict avoidance, and family involvement at mealtimes) between the pre-test and post-test periods. As shown in Table 5, there were significant changes in children's food fussiness scores, such that scores were higher at post-test (indicating an increase in food fussiness), and in stress/conflict avoidance and shared family food goals, such that parents expressed a desire for the family to avoid stress and to eat the same food at mealtimes more strongly at post-test. There were no significant changes in the goal relating to family involvement at mealtimes.

**Table 5:** Pre-test and post-test ratings of food fussiness and family mealtime goals (*N* = 47).

**Measure (possible score range)**	**Pre-test**	**Post-test**	**Pre–post comparison**
Food fussiness (6–30)	16.38 (5.86)	17.81 (5.02)	*t*(46) = −2.59, *p* = 0.013
**Family mealtime goals**			
Shared family food (1–5)	4.08 (0.75)	4.36 (0.69)	*t*(46) = −2.68, *p* = 0.010
Stress/conflict avoidance (1–5)	4.16 (0.68)	4.35 (0.67)	*t*(46) = −2.25, *p* = 0.030
Family involvement at mealtimes (1–5)	3.69 (0.71)	3.83 (0.71)	*t*(46) = −1.57, *p* = 0.124

## Discussion

Results of the present study suggest that giving parents a vegetable e-book to read with their children promoted more positive attitudes toward two vegetables that children were initially not keen on. After 2 weeks of reading an e-book about one of the two chosen vegetables, children were more willing to taste both the target and the non-target vegetable and consume them in larger portion sizes. Children were also perceived by their parents to like the two vegetables more.

Given previous findings of studies that have used physical books about vegetables (Houston-Price et al., 2009a,b, 2019; Heath et al., 2014; Barnes and Warren, 2017; Owen et al., 2018), we expected positive changes in children's attitudes to the vegetable shown in their e-book. As in previous studies, participants read the e-books as instructed, with an average of eight times over the two-week period, and they reported that both they and their child found the e-book enjoyable to read. These results support previous suggestions that both increased visual familiarity and the positive social context around families' interactions with the books may affect children's attitudes to the content of the book, supporting increases in willingness to taste, intake, and liking of the target vegetable. The increase in the average scores of willingness to taste mirrors a remarkable shift in children's attitude toward food, as after the intervention they were more likely to put the vegetables on their lips, while before they just touched them. A similar improvement emerged in the liking scores, showing that the vegetables were less detested.

However, the finding that these positive effects were not specific to the vegetable featured in the e-book was unexpected. Changes in willingness to taste, intake, and liking were also demonstrated for a second vegetable chosen by parents at the beginning of the study, which was not shown in the e-book. This finding stands in contrast to those of most other familiarization-based interventions conducted among preschoolers, where increased intake and liking is specific to the target vegetable (Remington et al., 2012; Fildes et al., 2014; Heath et al., 2014; Holley et al., 2015; Owen et al., 2018).

We interpret the pattern of results found in the current study as demonstrating a generalization effect of repeated exposure to one vegetable to another vegetable. This is a positive outcome, in the sense that reading an e-book about one vegetable was powerful enough to increase children's consumption and liking of another vegetable. A similar effect was reported by de Wild et al. (2017), who found an increase in spinach consumption among 2–4-year-old children who were repeatedly exposed to green beans as a control condition. Notably, the design of the de Wild et al. (2017) differed to that of the current study because they asked families to offer children repeated tastes of either spinach or green beans, while the current study relied on repeated visual exposures via e-books. Nevertheless, de Wild et al. (2017) attributed their carryover effect to the visual similarity between spinach and green beans, specifically their green color. However, visual similarity alone cannot fully explain the generalization effect observed in the current study, given that families were free to choose two vegetables from a list of 24 vegetables that varied in size, shape, and color, and many parents selected two foods that were visually quite dissimilar.

We therefore offer some tentative speculations about other factors that might have contributed to the generalization effect in this study. Parents' awareness that the e-book was intended to encourage their children to eat vegetables appears to have prompted them to offer their child the vegetables during meals during the reading period, even though they were not asked to do so. Although we do not know exactly how many times parents offered the two vegetables during this period, the large majority offered both the target vegetable (97%) and the non-target vegetable (84%) at least once. It appears that the mere act of identifying two vegetables that a child does not like, and being provided with an e-book about one of these, prompted parents to offer their both disliked vegetables, leading to changes in children's food preferences. It is also worth noting that those families who did not spontaneously offer both vegetables during the first post-test round agreed to do so within a subsequent 2-week period, before completing the post-test questionnaires. Thus, by the time the post-test questionnaires were collected, all families had offered both disliked vegetables, at least once but possibly more frequently. We therefore suggest that our results reflect the greater availability of and greater opportunities to taste both disliked vegetables that arose either spontaneously as a result of reading the e-book or following a gentle prompt by the research team.

Another interesting and unexpected finding was that children's food fussiness scores increased over time. Although it should be noted that mean scores corresponded only to an average level of food fussiness, meaning that children in the current sample exhibited fussy behaviors only sometimes, the improvement may well reflect parents' increased offering of disliked vegetables during the intervention, which would have provided them with opportunities to observe their child's aversive reactions toward these foods. There were also significant changes in parents' endorsement of stress/conflict avoidance and shared family food goals between pre-test and post-test, suggesting that participating in the e-book intervention increased parents' desire to prepare food that all members of the family would eat and desire for meals to be consumed in a relaxed atmosphere. These desires may be linked to children's increased food fussiness during the intervention. But it is equally plausible that participation in the study, and reading about healthy foods' origins and provenance, may have reminded parents of the importance of family mealtimes to them—an occasion that is crucial within Italian culture. It is noteworthy that the importance attributed by our sample to consuming the same food during meals is consistent with data showing that one-third of parents chose vegetables that other family members ate as well.

### Limitations and Future Directions

The results from this sample have limited generalizability to other Italian families because the study used a convenience sampling method to recruit participants. Nonetheless, analyses were run only after having reached the minimum number of subjects needed to detect significant effects in two key outcome measures (willingness to taste and liking); however, it was underpowered for intake, according to preliminary power calculations. Results related to increases in children's consumption of the vegetables should then be taken with caution. Future studies may clarify whether the observed changes in intake are robust in an analysis with greater power. Nevertheless, the statistical analysis used to evaluate our confirmatory hypotheses was faithful to our preregistered protocol, increasing the trustworthiness of the results.

The collected measures were parent-reported; therefore, the reported increases in children's attitudes toward vegetables may be due to parents reporting on socially desirable outcomes of the intervention. Nevertheless, scales used to measure willingness to taste and intake featured concrete behavioral indicators (e.g., put food in lips but not in mouth; a teaspoon), which served to reduce the subjectiveness of these ratings. Videotaped observations or weighing the food consumed by children can reduce the risk of social desirability; however, the awareness that they are being directly observed and assessed may lead to larger than expected changes in parent and child behavior.

The intervention relied on the principle of repeated visual exposures to increase children's familiarity with disliked foods. There was wide variability among families in the number of times they read the e-book (2–15 times) and how long they read it per occasion (2–20 mins). There is no consensus on the necessary number of visual exposures to produce changes in children's eating behaviors; though previous studies using different designs have resulted in a range of 2–8 (Rioux et al., 2018) and 6 to up to 40–50 exposures (Heath et al., 2014). No data was collected to examine why some parents read more or longer with their children than others. There are several competing possibilities, such as age-related differences in book reading behaviors, time constraints, lack of interest, or family book reading habits. Conversely, it is possible that parents have successfully introduced the target vegetable to the child after only a few visual exposures. Future studies can investigate individual differences in intervention uptake or set precise targets on the minimum number of exposures to be made.

A limitation of the present study is that the study design only involved a within-subjects comparison (pre versus post-test; exposed versus non-exposed vegetable). Further research might examine the effects of e-book reading by including picture book reading as an active control group and a no-reading group as a passive control group. This would help address whether e-books are equally effective or have a larger impact on children's eating behavior as traditional picture books or no reading at all.

Another limitation of the study is that it is unable to examine the unique potential of e-books over traditional picture books; specifically through the ease with which one can personalize the vegetable e-book. Although some families were informed of the additional editing features of the *Our Story 2* app, most parents found the e-book sufficient as it was and felt that modifications were not necessary. Future studies can be designed to investigate the profiles of families who choose to use additional app features and any differences in children's eating-related outcomes among the families who do. Cross-cultural comparisons may also uncover differences in evaluation outcomes across samples or between countries, in terms of the effectiveness of vegetable e-books and whether effects generalize to non-targeted foods in different populations. Finally, improvements should be made to the usability of the *Our Story 2* app and its compatibility with different tablet and mobile devices to expand the accessibility of the See & Eat e-books to broader audiences.

Despite these limitations, the results of the present study suggest that an e-book intervention could be a cheap and easy-to-disseminate method of influencing children's food preferences via repeated visual exposure to vegetables, at least among families that have access to an appropriate electronic device.

## Data Availability Statement

The raw data supporting the conclusions of this article will be made available by the authors, without undue reservation.

## Ethics Statement

The studies involving human participants were reviewed and approved by University of Reading, Coordinator for Quality Assurance in Research (UREC Secretary). Written informed consent to participate in this study was provided by the participants' legal guardian/next of kin.

## Author Contributions

CH-P, DB, GC, KMD, MC, NAM, and PM: conceptualization, methodology. MC and MF: data curation. KMD and MC: formal analysis. KMD and MC: writing—original draft. CH-P, DB, NAM, and PM: writing—review and editing. GC: supervision. All authors contributed to the article and approved the submitted version.

## Conflict of Interest

The authors declare that the research was conducted in the absence of any commercial or financial relationships that could be construed as a potential conflict of interest.

## Publisher's Note

All claims expressed in this article are solely those of the authors and do not necessarily represent those of their affiliated organizations, or those of the publisher, the editors and the reviewers. Any product that may be evaluated in this article, or claim that may be made by its manufacturer, is not guaranteed or endorsed by the publisher.
