# Corrigendum: See & Eat! Using E-books to Promote Vegetable Eating Among Preschoolers: Findings From an Italian Sample

**DOI:** 10.3389/fpsyg.2021.773658

**Published:** 2021-10-19

**Authors:** Marcella Caputi, Katrina May Dulay, Daniela Bulgarelli, Carmel Houston-Price, Giuseppina Cerrato, Mauro Fanelli, Natalie A. Masento, Paola Molina

**Affiliations:** ^1^Department of Psychology, Università degli Studi di Torino, Torino, Italy; ^2^Sigmund Freud University, Milano, Italy; ^3^School of Psychology and Clinical Language Sciences, University of Reading, Reading, United Kingdom; ^4^Department of Education, University of Oxford, Oxford, United Kingdom; ^5^Department of Chemistry, Università degli Studi di Torino, Torino, Italy; ^6^Interuniversity Department of Regional and Urban Studies and Planning, Università degli Studi di Torino, Torino, Italy

**Keywords:** vegetable intake, visual familiarity, visual exposure, healthy eating, food fussiness, mealtime goal

In the published article, there was an error in the affiliation superscript of Daniela Bulgarelli. Instead of “2^*^”, it should be “1^*^”.

The authors apologize for this error and state that this does not change the scientific conclusions of the article in any way. The original article has been updated.

## Publisher's Note

All claims expressed in this article are solely those of the authors and do not necessarily represent those of their affiliated organizations, or those of the publisher, the editors and the reviewers. Any product that may be evaluated in this article, or claim that may be made by its manufacturer, is not guaranteed or endorsed by the publisher.

